# Fli1 deficiency suppresses RALDH1 activity of dermal dendritic cells and related induction of regulatory T cells: a possible role in scleroderma

**DOI:** 10.1186/s13075-021-02520-z

**Published:** 2021-05-08

**Authors:** Shunsuke Miura, Yusuke Watanabe, Ryosuke Saigusa, Takashi Yamashita, Kouki Nakamura, Megumi Hirabayashi, Takuya Miyagawa, Ayumi Yoshizaki, Maria Trojanowska, Shinichi Sato, Yoshihide Asano

**Affiliations:** 1 Department of Dermatology, University of Tokyo Graduate School of Medicine, 7-3-1 Hongo, Bunkyo-ku, Tokyo, 113-8655 Japan; 2 Arthritis Center, Boston University School of Medicine, Boston, MA USA

**Keywords:** Aldehyde dehydrogenase 1 family member A1, Dermal dendritic cells, Fli1, Regulatory T cells, Systemic sclerosis

## Abstract

**Background:**

Aldehyde dehydrogenase 1 family member A1 (RALDH1)-producing dermal dendritic cells (DCs), a conventional DC subset regulating skin fibrosis, are decreased in the involved skin of patients with systemic sclerosis (SSc). In this study, we investigated the contribution of Fli1 deficiency, a potential predisposing factor of SSc, to the phenotypical alteration of RALDH1-producing dermal DCs by using SSc model mice and SSc skin samples.

**Methods:**

Bleomycin (BLM)-induced skin fibrosis was generated with *Fli1*^+/−^ and wild-type mice. The proportions of DC and CD4^+^ T cell subsets were determined by flow cytometry in the dermis of BLM-treated mice. Fli1 expression in dermal DCs was evaluated by immunofluorescence with skin samples of SSc and healthy control subjects.

**Results:**

RALDH activity of dermal DCs was significantly decreased in BLM-treated *Fli1*^+/−^ mice compared with BLM-treated wild-type mice, whereas the proportion of CD103^−^CD11b^−^ dermal DCs, a major DC subset producing RALDH1 in response to BLM injection, was comparable between groups. Relevant to this finding, the proportion of regulatory T cells (Tregs) in the dermis was decreased in BLM-treated *Fli1*^+/−^ mice relative to BLM-treated wild-type mice, while the proportions of Th1, Th2 and Th17 cells were unaltered. In the involved skin of SSc patients, Fli1 was downregulated in CD11c^+^ cells, including dermal DCs.

**Conclusions:**

Fli1 deficiency inhibits RALDH1 activity of CD103^−^CD11b^−^ dermal DCs and related induction of Tregs in BLM-treated mice. Considering Fli1 reduction in SSc dermal DCs, Fli1deficiency may impair the dermal DC-Treg system, contributing to the development of skin fibrosis in SSc.

**Supplementary Information:**

The online version contains supplementary material available at 10.1186/s13075-021-02520-z.

## Background

Systemic sclerosis (SSc) is a multisystem autoimmune disease characterized by vasculopathy and fibrosis of the skin and certain internal organs [1–3]. Although its etiology remains enigmatic, skin fibrosis is believed to be caused by the complex, but orchestrated interaction of interstitial fibroblasts with immune cells, vascular cells, keratinocytes, and adipocytes [4–6]. Among immune cells, previous research attention has focused on B cells, T cells, and macrophages in SSc [7]. Regarding dendritic cells (DCs), recent studies have revealed the contribution of plasmacytoid DCs (pDCs) to the development of SSc [8], but the role of other DC subsets, such as conventional DCs (cDCs) and monocyte-derived DCs, had remained unknown. However, our recent study identified aldehyde dehydrogenase 1 family member A1 (ALDH1A1/RALDH1)-producing CD103^−^CD11b^−^ cDCs as a critical DC subset regulating the development of inducible Foxp3^+^ regulatory T cells (Tregs) in BLM-treated mice and demonstrated decreased numbers of RALDH1-producing dermal DCs in SSc involved skin [9]. Since dermal DCs serve as a key mediator of skin immunity [10], further characterization of RALDH1-producing dermal DCs would enable us to better understand the pathogenesis of SSc.

In the skin, DCs and macrophages are the principal antigen presenting cells. Langerhans cells in the epidermis and cDCs in the dermis uptake internal and external antigens, migrate to lymph nodes, and present them to naïve T cells, thus serving as a bridge between innate and adaptive immunity. Under physiological conditions, a fraction of cDCs undergoes homeostatic maturation, then transport cutaneous self-antigen to the T cell zones of draining lymph nodes and activate an abortive program of autoreactive T cells. In contrast, when encountering external antigen, cDCs experience terminal differentiation, migrate to draining lymph nodes, and promote clonal expansion of naïve antigen-specific T cells and the acquisition of T cell effector functions. During this highly organized process, cDCs regulate Treg development from conventional naïve CD4^+^ T cells by producing retinoic acid [11]. Retinoic acid is generated from vitamin A through a sequential step tightly regulated by three enzymes, namely, alcohol dehydrogenase, retinol dehydrogenase, and aldehyde dehydrogenase 1 (ALDH1/RALDH). Although the former two enzymes are ubiquitously produced in various cell types, RALDH is exclusively expressed in certain cell types, including cDCs. Thus, RALDH-producing cDCs promote retinoic acid-dependent Treg development together with a submitogenic dose of antigen, low costimulation, and high levels of transforming growth factor (TGF)-β [12–15]. In bleomycin (BLM)-induced skin fibrosis, CD103^−^CD11b^−^ dermal cDCs express RALDH1 and promote Treg development [9]. Importantly, the number of RALDH1-producing dermal cDCs is decreased in the involved skin of SSc patients and tends to correlate with the severity of skin fibrosis [9], suggesting that retinoic acid-dependent induction of Tregs by cDCs is involved in the development of SSc-associated skin fibrosis.

Friend leukemia virus integration 1 (Fli1), a member of Ets transcription factor family, is broadly downregulated in various types of cells in the skin of SSc patients [16]. Among numerous disease-associated molecules, Fli1 is the only one whose expression is shown to be regulated by epigenetic and genetic mechanisms; the CpG island of the *FLI1* promoter is hypermethylated in the involved skin of SSc patients, and extended repeat alleles of *FLI1* (GA)_*n*_ microsatellite are associated with lower *FLI1* mRNA levels and susceptibility to SSc [17, 18]. Fli1 deficiency seems to be a potent predisposing factor of SSc due to the following reasons; (i) Fli1 deficiency induces SSc-like phenotypes in dermal fibroblasts, endothelial cells, macrophages, and keratinocytes [19–29]; (ii) Fli1 heterozygous deficiency enhances SSc-like phenotypes in dermal fibroblasts, endothelial cells, and macrophages in mice treated with BLM [30]; (iii) endothelial cell-specific *Fli1* knockout mice resemble vascular structural and functional abnormalities characteristic of SSc vasculopathy [31]; (iv) bosentan, a dual endothelin receptor antagonist that prevents the development of new digital ulcers, increases endothelial Fli1 expression in SSc patients [32]; and (v) epithelial cell-specific *Fli1* knockout mice develop dermal and esophageal fibrosis characterized by the activation of stratified squamous epithelia and interstitial lung disease due to the downregulation of the autoimmune regulator (AIRE) in the thymus, recapitulating selective organ fibrosis of SSc [33]. Thus, Fli1 deficiency-related studies have provided new insights into the pathogenesis of SSc.

Based on these backgrounds, we investigated the contribution of Fli1 deficiency to the phenotypical alteration of dermal DCs in the context of SSc and related conditions by using BLM-treated *Fli1*^+/−^ mice and SSc skin samples.

## Methods

### A BLM-induced murine SSc model

BLM (200 μg, Nippon Kayaku, Tokyo, Japan) dissolved in phosphate-buffered saline (PBS) or control PBS was injected subcutaneously into a single location on the back of 8-week-old female wild-type (WT) mice (C57BL/6) and *Fli1*^+/−^ mice daily. The injection was conducted consecutively for 1 week.

### Flow cytometry

The day following the completion of 1-week BLM injection, lymphocytes from draining lymph nodes and the dermis of the lower back skin were obtained. In the surface staining, cells were stained with antibodies against CD11b (M1/70), CD11c (N418), and CD103 (2E7; all from BioLegend, San Diego, CA, USA). In intracellular cytokine staining, cells were stimulated with 10 ng/ml phorbol myristate acetate (Sigma-Aldrich, St. Louis, MO) and 1 μg/ml ionomycin (Sigma-Aldrich) in the presence of 1 μg/ml brefeldin A (GolgiStop; BD PharMingen) for 4 h. Cells were washed, stained for the surface markers CD4 (RM4-5) and CD25 (PC61; all from BioLegend), treated with fixation/permeabilization working solution (BD PharMingen), and then stained with anti-interleukin (IL)-4 (11B11), anti-IL-17A (TC11.18H10), and anti-interferon (IFN)-γ (XMG1.2; all from BioLegend) antibodies. To analyze transcription factors, antibody against Foxp3 (FJK-16 s; eBioscience, San Diego, CA, USA) were used. Cells were analyzed on a FACSVerse flow cytometer (BD Biosciences, San Diego, CA, USA). The populations of positive and negative cells were determined using nonreactive isotype-matched antibodies as controls. Gating strategies are shown in supplementary Fig. S1.

### Analysis of aldehyde dehydrogenase (ALDH) activity at the single-cell level

The presence of cells displaying ALDH activity was determined using an ALDEFLUOR staining kit (StemCell Technologies, Vancouver, BC, Canada). The following modifications were introduced into the manufacturer’s protocol. Briefly, cells (1 × 10^6^ cells/mL) were incubated in the dark for 45 min at 37 °C in ALDEFLUOR assay buffer containing activated ALDEFLUOR substrate, with or without the ALDH inhibitor diethylaminobenzaldehyde (DEAB). Cells were subsequently stained using the specified antibodies in ice-cold ALDEFLUOR assay buffer. Cells were subsequently washed in ALDEFLUOR assay buffer, resuspended in ALDEFLUOR assay buffer, and kept on ice before analysis on a FACSVerse flow cytometer (BD Biosciences).

### Immunofluorescence

Mouse anti-CD11c antibody (Santa Cruz Biotechnology, Dallas, TX, USA) and rabbit anti-Fli1 antibody (Santa Cruz Biotechnology) were used as primary antibodies, and Alexa Fluor 555-conjugated donkey anti-mouse IgG antibody (Thermo Fisher Scientific, Waltham, MA, USA) and fluorescein isothiocyanate-conjugated donkey anti-rabbit IgG antibody (Santa Cruz Biotechnology) were used as secondary antibodies for human skin sections. Coverslips were mounted by Vectashield with DAPI (Vector Laboratories, Burlingame, CA, USA), and staining was examined with Biozero BZ-8000 (Keyence, Osaka, Japan) at 495 nm (green), 565 nm (red), and 400 nm (blue). Ten random grids were evaluated under high magnification by 2 independent researchers in a blinded manner.

### Statistical analysis

Statistical analysis was carried out using GraphPad Prism 8.30 (GraphPad Software, San Diego, CA, USA). The Mann-Whitney *U* test was used for two-group comparison. The Kruskal-Wallis test followed by the Dunn’s multiple comparison test was used for three-group multiple comparison. Statistical significance was defined as a *P* value of < 0.05.

## Results

### Fli1 heterozygous deficiency suppresses the activity of ALDH in CD103^−^CD11b^−^ dermal DCs in BLM-treated mice

**Fig. 1:**
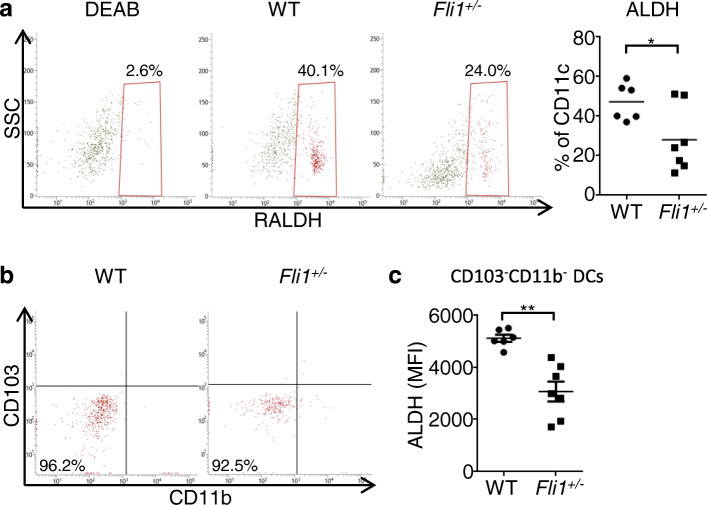
Fli1 heterozygous deficiency suppresses the activity of ALDH in CD103^−^CD11b^−^ dermal DCs in BLM-treated mice. Wild-type (WT) and *Fli1*^*+/*−^ mice were treated with bleomycin (BLM) for a week. **a** Representative flow cytometry plots of ALDH staining in CD11c^+^ cells from the dermis of BLM-treated WT and *Fli1*^*+/*−^ mice are shown (left panels). The result in the presence of the ALDH inhibitor diethylaminobenzaldehyde (DEAB) is shown as control. The proportions of CD11c^+^ cells positive for ALDH are summarized in a right graph (*n* = 6–7 per group). **b** Representative flow cytometry plots of CD11b and CD103 staining in ALDH-producing CD11c^+^ cells from the dermis of BLM-treated WT and *Fli1*^*+/*−^ mice are shown (*n* = 6–7 per group). **c** Mean fluorescence intensity (MFI) of ALDH was examined in CD11b^−^CD103^−^ DCs isolated from the dermis. Each graph indicates mean ± SEM of the indicated parameters. * *P* < 0.05; ** *P* < 0.01

As an initial experiment, we focused on the RALDH1 activity in dermal DCs of *Fli1*^+/−^ and WT mice because RALDH1 is a key enzyme regulating the induction of Tregs in BLM-treated mice [9]. To this end, we employed an ALDEFLUOR staining kit in which we can evaluate the summation of ALDH activities. At the time of writing, 17 different ALDH family proteins have been identified [34]. Among them, only the RALDH family has been confirmed to be expressed in DCs [15]. Therefore, when we apply this method to DCs, we can evaluate the summation of RALDH1, RALDH2, and RALDH3 activities. As shown in Fig. 1a, b, the number of dermal DCs exhibiting ALDH activity was decreased in BLM-treated *Fli1*^+/−^ mice compared with BLM-treated WT mice, and the majority of dermal DCs with ALDH activity were CD103^−^CD11b^−^ DCs in both strains. Furthermore, mean fluorescence intensity of ALDH activity was significantly lower in CD103^−^CD11b^−^ DCs of BLM-treated *Fli1*^+/−^ mice than in those of BLM-treated WT mice (Fig. 1c). Given that BLM selectively induces RALDH1 activity as demonstrated in our previous study [9], these results indicate that Fli1 heterozygous deficiency suppresses the activity of RALDH1 in CD103^−^CD11b^−^ dermal DCs after the injection of BLM.

### The impact of Fli1 deficiency on the proportion of dermal DC subsets

**Fig. 2:**
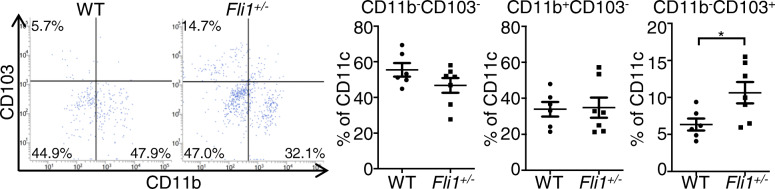
The impact of Fli1 deficiency on the proportion of dermal DC subsets. Wild-type (WT) and *Fli1*^*+/*−^ mice were treated with bleomycin (BLM) for a week. Representative flow cytometry plots of CD11b and CD103 staining in CD11c^+^ cells from the dermis are shown. The percentages of CD11b^−^CD103^−^, CD11b^+^CD103^−^, and CD11b^−^CD103^+^ cells in CD11c^+^ cells in WT and/or *Fli1*^*+/*−^ mice are summarized in graphs. Flow cytometry profiles are representative of 6–7 individual animals from at least 2 separate experiments. Bars show the mean ± SEM. *P < 0.05

Generally, dermal DCs are classified into CD103^+^ DCs, CD103^−^CD11b^−^ DCs and CD103^−^CD11b^+^ DCs in mice; the former two subsets are cDCs, but CD103^−^CD11b^+^ DCs comprise cDCs, monocyte-derived DCs, and macrophages [10]. Therefore, we compared the proportions of these dermal DC subsets in the dermis of BLM-treated *Fli1*^+/−^ and WT mice. In the dermis, the proportion of CD103^+^ DCs was significantly increased, while the proportions of CD103^−^CD11b^+^ DCs and CD103^−^CD11b^−^ DCs were unaltered in BLM-treated *Fli1*^+/−^ mice compared with BLM-treated WT mice (Fig. 2). Taken together with the results in Fig. 1, these results indicate that the decreased RALDH1 activity is not due to the decreased proportion of CD103^−^CD11b^−^ DCs. Therefore, RALDH1-producing CD103^−^CD11b^−^ dermal cDC-associated biological events are likely inhibited in the dermis of BLM-treated *Fli1*^+/−^ mice.

### The proportion of Tregs is decreased in the dermis of BLM-treated *Fli1*^+/−^ mice

**Fig. 3:**
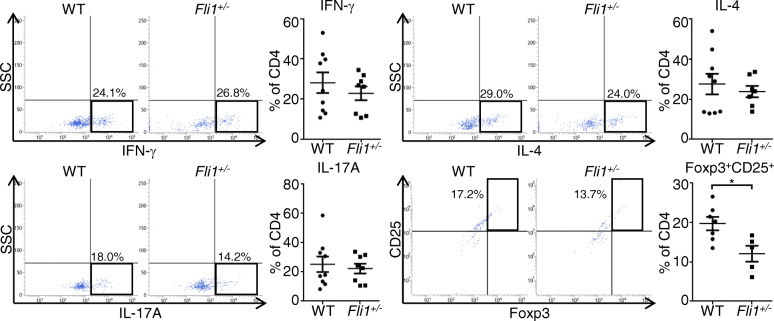
The proportion of Tregs is decreased in the dermis of BLM-treated *Fli1*^+/−^ mice. Wild-type (WT) and *Fli1*^*+/*−^ mice were treated with bleomycin (BLM) for a week. The percentages of IFN-γ-, IL-4-, and IL-17A-producing CD4^+^ T cells and CD4^+^CD25^+^Foxp3^+^ Tregs in the dermis were determined by flow cytometry. Flow cytometry profiles are representative of 5-9 individual animals from at least 2 separate experiments. Each graph indicates mean ± SEM of the indicated parameters. *P < 0.05

To determine if Treg induction is disturbed in BLM-treated *Fli1*^+/−^ mice, we assessed the proportions of CD4^+^ T cell subsets, such as Th1, Th2, Th17, and Tregs, in the dermis of BLM-treated *Fli1*^+/−^ and WT mice. As shown in Fig. 3, the proportion of Tregs was significantly decreased in BLM-treated *Fli1*^+/−^ mice compared with BLM-treated WT mice, while the proportions of Th1, Th2, and Th17 cells revealed similar results in the two groups. Taken together with the results in Fig. 2, these results suggest that RALDH1-producing CD103^−^CD11b^−^ dermal cDC-dependent induction of Tregs is inhibited in the dermis of BLM-treated *Fli1*^+/−^ mice.

### Fli1 is downregulated in dermal DCs of SSc patients

**Fig. 4:**
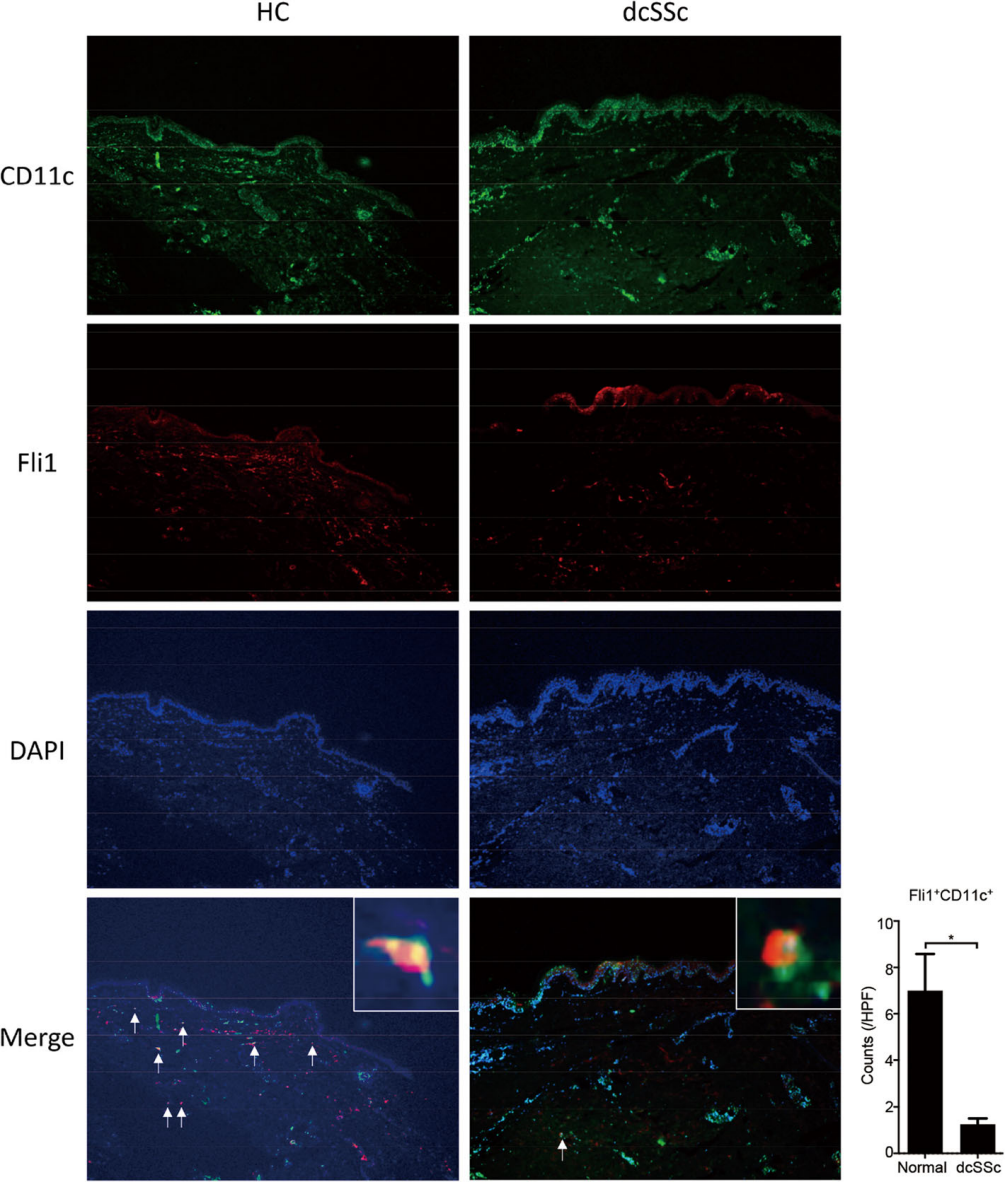
Fli1 is downregulated in dermal DCs of SSc patients. Immunofluorescence with antibodies against CD11c and Fli1 was conducted with skin samples from diffuse cutaneous systemic sclerosis (dcSSc) and healthy control subjects. Representative immunofluorescence images are shown (photo panels). Double positive cells were counted in a × 100 high-power field (HPF; right graph; *n* = 4, each). Graph indicates mean ± SEM. Arrows indicate double positive cells. Scale bars are 100 μm. *P < 0.05

Finally, we explored the expression of Fli1 in dermal DCs in SSc patients. To this end, we carried out double immunofluorescence for CD11c and Fli1. As shown in of Fig. 4, we could see CD11c/Fli1 double positive cells in the dermis of human skin samples. Of note, the number of double positive cells was significantly decreased in diffuse cutaneous SSc patients compared with healthy controls (bottom panels of photos and a right panel in Fig. 4), while the number of CD11c-positive cells was slightly increased in diffuse cutaneous SSc patients, reflecting chronic inflammation (top panels of photos in Fig. 4). These results indicate that Fli1 expression is decreased in dermal CD11c^+^ cells, including dermal DCs, in SSc lesional skin. Given the decreased number of RALDH1-expressing dermal DCs in diffuse cutaneous SSc patients compared with healthy controls [9], Fli1 deficiency may contribute to the RALDH1 suppression in dermal DCs of diffuse cutaneous SSc patients.

## Discussion

Previous clinical and experimental studies on the role of DCs in SSc development have been predominantly focused on pDCs that contribute to sterile inflammation related to autoimmune rheumatic disorders through type I interferon (IFN-I) production in response to self-nucleic acids [8]. Indeed, the IFN signature has been observed in the peripheral blood, skin, and lungs of SSc patients [35, 36], and circulating SSc pDCs secrete elevated levels of IFN-I due to the aberrantly expressed Toll-like receptor (TLR) 8 as well as TLR7 and TLR9. Importantly, pDC depletion not only prevents the development of skin fibrosis, but also ameliorates established skin fibrosis in BLM-treated mice [37]. Thus, it is currently accepted that pDCs play a critical role in SSc pathogenesis. On the other hand, although cDCs act as a critical regulator of skin immunity [10], their roles in immunopathology of SSc have remained poorly understood.

In this study, we employed *Fli1*^+/−^ mice to investigate the phenotypical alteration of cDCs with relevance to SSc-associated disease pathology because BLM-treated *Fli1*^+/−^ mice exhibit SSc-like features in various cell types [30, 38]. Since BLM injection induces RALDH1 production in CD103^−^CD11b^−^ dermal cDCs and subsequently promotes Treg development in WT mice [9], the enhancement of BLM-dependent dermal fibrosis in *Fli1*^+/−^ mice suggests that Fli1 deficiency impairs the regulatory function of CD103^−^CD11b^−^ dermal cDCs. As expected, RALDH activity was decreased in CD103^−^CD11b^−^ dermal cDCs of BLM-treated *Fli1*^+/−^ mice. Furthermore, the Treg proportion was decreased in the dermis of BLM-treated *Fli1*^+/−^ mice. Although we could not exclude the possibility that Fli1 deficiency directly affects the differentiation of naïve CD4^+^ T cells to Tregs, these current results indicate that decreased RALDH1 activity of CD103^−^CD11b^−^ DCs at least partially contributes to the dysregulated Treg induction in the skin of BLM-treated *Fli1*^+/−^ mice. This notion is supported by our previous finding that *Raldh1* siRNA suppresses Treg induction in the skin of BLM-treated WT mice [9]. Considering decreased expression of Fli1 and RALDH1 in CD11c^+^ cells as shown in our previous and current studies and exclusive expression of RALDH in cDCs [9, 11], we can plausibly conclude that Fli1 deficiency induces SSc-like phenotypes in dermal DCs, in addition to previously shown fibroblasts, endothelial cells, macrophages and epithelial cells [22, 30, 31, 33, 39, 40].

The alteration of T cell subsets has been well studied in the field of SSc research. Generally, the balances of Th1/Th2 and Th17/Treg immune responses skew toward Th2 and Th17 predominance [41–44], and Treg function is impaired in the active stage of SSc [45]. In addition, the proportion of Th2-like Tregs is increased in the involved skin of SSc patients [46]. Importantly, these characteristic alterations of CD4^+^ T cell balance are reproduced in BLM-treated *Fli1*^+/−^ mice [30], and some of the potential underlying mechanisms have been shown. For instance, Fli1 deficiency induces the expression of IL-33 and galectin-9 in dermal fibroblasts, resulting in the promotion of Th2-like Treg induction and the suppression of Th1 development, respectively [30, 38]. Also, Fli1 deficiency facilitates IL-6 production in dermal fibroblasts, promoting Th17 differentiation together with active TGF-β and IL-1β that are elevated in the skin of BLM-treated *Fli1*^+/−^ mice [38]. The current study added a new insight into the contribution of Fli1 deficiency to the induction of SSc-like CD4^+^ T cell balance, namely, the decrease in Treg proportion due to the reduced RALDH1 expression in CD103^−^CD11b^−^ dermal cDCs. Thus, Fli1 deficiency may serve as a key regulator of skin inflammation, as well as skin fibrosis and vasculopathy, in SSc.

The current study suggests that administration of retinoic acid is a potential therapeutic strategy to increase the proportion of Tregs in SSc [47]. However, it has been long recognized that overexposure to retinoic acid causes widespread teratogenesis in humans [48]. An alternative therapeutic strategy is to restore Fli1 expression in cDCs, possibly resulting in increased RALDH1 activity of cDCs and subsequent induction of Tregs in the involved skin of SSc patients. From this perspective, it is quite important to identify the mechanism by which Fli1 is downregulated in SSc cDCs. In dermal fibroblasts and dermal microvascular endothelial cells, endothelin-1 stimulation suppresses Fli1 expression and induces SSc-like phenotypes [39, 40, 49]. Importantly, endothelin-1 (ET-1) directly induces the phenotypic maturation of bone marrow-derived DCs that preferentially prime T cells to produce Th17 cytokines [50]. Given that RALDH1-producing cDCs skew Th17/Treg balance toward Treg predominance [9, 47], ET-1 may be a possible factor suppressing RALDH1 production in cDCs, in which ET-1-dependent Fli1 suppression may be involved. Since bosentan, a dual antagonist of endothelin receptors, reverses SSc-like phenotypes of dermal microvascular endothelial cells by increasing Fli1 expression and improves SSc vascular features at the molecular and morphological levels [32, 39, 40, 51–53], this reagent may modulate skin immunity of SSc patients. Further studies are required to clarify this point.

The limitation of this study is the lack of data on detailed mechanism by which RALDH1 expression is regulated by Fli1 in CD103^−^CD11b^−^ dermal DC. To address this issue, further studies are required to investigate whether Fli1 directly regulates the transcriptional activity of RALDH1 in dermal DCs. Of interest, Yashiro et al. [54] demonstrated the transcriptional regulation of RALDH2 expression by PU.1, a member of Ets transcription factor family, in bone marrow-derived DCs of BALB/c mice. These current and previous findings support the canonical idea that the Ets transcription factor family plays a critical part in the development and differentiation of hematopoietic cells.

## Conclusion

This is the first report demonstrating the critical role of Fli1 deficiency in CD103^−^CD11b^−^ dermal cDC-dependent induction of Tregs in BLM-treated mice, an established SSc animal model. The current data indicate that dermal DCs are yet another cell type in which Fli1 deficiency induces an SSc-like phenotype. Although further studies are required to clarify the role of Fli1 deficiency in human cDCs, this study strengthens the notion that Fli1 deficiency serves as a potential predisposing factor of SSc.

## Supplementary Information

**Additional file 1: Supplementary Figure 1.** The gating strategies of Figure 1B, Figure 2 and Figure 3. A. Representative ancestor gates of Figure 1B and Figure 2. B. Representative ancestor gates of Figure 3.

## Data Availability

Not applicable.

## Abbreviations

SSc: Systemic sclerosis
DCs: Dendritic cells
pDCs: Plasmacytoid DCs
cDCs: Conventional DCs
ALDH1A1/RALDH1: Aldehyde dehydrogenase 1 family member A1
Tregs: Regulatory T cells
ALDH1/RALDH: Aldehyde dehydrogenase 1
TGF: Transforming growth factor
Fli1: Friend leukemia virus integration 1
BLM: Bleomycin
AIRE: Autoimmune regulator
PBS: Phosphate-buffered saline
WT: Wild-type
ALDH: Aldehyde dehydrogenase
DEAB: Diethylaminobenzaldehyde
IFN-I: Type I interferon
TLR: Toll-like receptor
ET-1: Endothelin-1

## References

[1] Denton CP, Khanna D (2017). Systemic sclerosis. Lancet.

[2] Allanore Y, Simms R, Distler O, Trojanowska M, Pope J, Denton CP, Varga J (2015). Systemic sclerosis. Nat Rev Dis Primers.

[3] Asano Y (2018). Systemic sclerosis. J Dermatol.

[4] Aden N, Nuttall A, Shiwen X, de Winter P, Leask A, Black CM, Denton CP, Abraham DJ, Stratton RJ (2010). Epithelial cells promote fibroblast activation via IL-1alpha in systemic sclerosis. J Invest Dermatol.

[5] Asano Y, Sato S (2015). Vasculopathy in scleroderma. Semin Immunopathol.

[6] Varga J, Marangoni RG (2017). Systemic sclerosis in 2016: dermal white adipose tissue implicated in SSc pathogenesis. Nat Rev Rheumatol.

[7] Asano Y, Varga J (2020). Rationally-based therapeutic disease modification in systemic sclerosis: novel strategies. Semin Cell Dev Biol.

[8] Carvalheiro T, Zimmermann M, Radstake T, Marut W (2020). Novel insights into dendritic cells in the pathogenesis of systemic sclerosis. Clin Exp Immunol.

[9] Miura S, Asano Y, Saigusa R, Yamashita T, Taniguchi T, Takahashi T, Ichimura Y, Toyama T, Yoshizaki A, Sato S, Kadono T (2020). Regulation of skin fibrosis by RALDH1-producing dermal dendritic cells via retinoic acid-mediated regulatory T cell induction: a role in scleroderma. J Dermatol Sci.

[10] Malissen B, Tamoutounour S, Henri S (2014). The origins and functions of dendritic cells and macrophages in the skin. Nat Rev Immunol.

[11] Niederreither K, Dolle P (2008). Retinoic acid in development: towards an integrated view. Nat Rev Genet.

[12] Coombes JL, Siddiqui KR, Arancibia-Carcamo CV, Hall J, Sun CM, Belkaid Y, Powrie F (2007). A functionally specialized population of mucosal CD103+ DCs induces Foxp3+ regulatory T cells via a TGF-beta and retinoic acid-dependent mechanism. J Exp Med.

[13] Sun CM, Hall JA, Blank RB, Bouladoux N, Oukka M, Mora JR, Belkaid Y (2007). Small intestine lamina propria dendritic cells promote de novo generation of Foxp3 T reg cells via retinoic acid. J Exp Med.

[14] Mucida D, Park Y, Kim G, Turovskaya O, Scott I, Kronenberg M, Cheroutre H (2007). Reciprocal TH17 and regulatory T cell differentiation mediated by retinoic acid. Science.

[15] Guilliams M, Crozat K, Henri S, Tamoutounour S, Grenot P, Devilard E, de Bovis B, Alexopoulou L, Dalod M, Malissen B (2010). Skin-draining lymph nodes contain dermis-derived CD103(-) dendritic cells that constitutively produce retinoic acid and induce Foxp3(+) regulatory T cells. Blood.

[16] Kubo M, Czuwara-Ladykowska J, Moussa O, Markiewicz M, Smith E, Silver RM, Jablonska S, Blaszczyk M, Watson DK, Trojanowska M (2003). Persistent down-regulation of Fli1, a suppressor of collagen transcription, in fibrotic scleroderma skin. Am J Pathol.

[17] Wang Y, Fan PS, Kahaleh B (2006). Association between enhanced type I collagen expression and epigenetic repression of the FLI1 gene in scleroderma fibroblasts. Arthritis Rheum.

[18] Yamashita K, Kawasaki A, Matsushita T, Furukawa H, Kondo Y, Okiyama N, Nagaoka S, Shimada K, Sugii S, Katayama M, Hirohata S, Okamoto A, Chiba N, Suematsu E, Setoguchi K, Migita K, Sumida T, Tohma S, Hamaguchi Y, Hasegawa M, Sato S, Kawaguchi Y, Takehara K, Tsuchiya N (2020). Association of functional (GA)n microsatellite polymorphism in the FLI1 gene with susceptibility to human systemic sclerosis. Rheumatology (Oxford).

[19] Ichimura Y, Asano Y, Akamata K, Noda S, Taniguchi T, Takahashi T, Toyama T, Tada Y, Sugaya M, Sato S, Kadono T (2015). Progranulin overproduction due to Fli-1 deficiency contributes to the resistance of dermal fibroblasts to tumor necrosis factor in systemic sclerosis. Arthritis Rheumatol.

[20] Saigusa R, Asano Y, Nakamura K, Hirabayashi M, Miura S, Yamashita T, Taniguchi T, Ichimura Y, Takahashi T, Yoshizaki A, Miyagaki T, Sugaya M, Sato S (2017). Systemic sclerosis dermal fibroblasts suppress Th1 cytokine production via galectin-9 overproduction due to Fli1 deficiency. J Invest Dermatol.

[21] Saigusa R, Asano Y, Taniguchi T, Yamashita T, Takahashi T, Ichimura Y, Toyama T, Tamaki Z, Tada Y, Sugaya M, Kadono T, Sato S (2015). A possible contribution of endothelial CCN1 downregulation due to Fli1 deficiency to the development of digital ulcers in systemic sclerosis. Exp Dermatol.

[22] Taniguchi T, Miyagawa T, Toyama S, Yamashita T, Nakamura K, Saigusa R, Ichimura Y, Takahashi T, Toyama T, Yoshizaki A, Sato S, Asano Y (2018). CXCL13 produced by macrophages due to Fli1 deficiency may contribute to the development of tissue fibrosis, vasculopathy and immune activation in systemic sclerosis. Exp Dermatol.

[23] Noda S, Asano Y, Takahashi T, Akamata K, Aozasa N, Taniguchi T, Ichimura Y, Toyama T, Sumida H, Kuwano Y, Yanaba K, Tada Y, Sugaya M, Kadono T, Sato S (2013). Decreased cathepsin V expression due to Fli1 deficiency contributes to the development of dermal fibrosis and proliferative vasculopathy in systemic sclerosis. Rheumatology (Oxford).

[24] Noda S, Asano Y, Akamata K, Aozasa N, Taniguchi T, Takahashi T, Ichimura Y, Toyama T, Sumida H, Yanaba K, Tada Y, Sugaya M, Kadono T, Sato S (2012). A possible contribution of altered cathepsin B expression to the development of skin sclerosis and vasculopathy in systemic sclerosis. PLoS One.

[25] Ichimura Y, Asano Y, Akamata K, Takahashi T, Noda S, Taniguchi T, Toyama T, Aozasa N, Sumida H, Kuwano Y, Yanaba K, Tada Y, Sugaya M, Sato S, Kadono T (2014). Fli1 deficiency contributes to the suppression of endothelial CXCL5 expression in systemic sclerosis. Arch Dermatol Res.

[26] Takahashi T, Asano Y, Noda S, Aozasa N, Akamata K, Taniguchi T, Ichimura Y, Toyama T, Sumida H, Kuwano Y, Tada Y, Sugaya M, Kadono T, Sato S (2015). A possible contribution of lipocalin-2 to the development of dermal fibrosis, pulmonary vascular involvement and renal dysfunction in systemic sclerosis. Br J Dermatol.

[27] Akamata K, Asano Y, Taniguchi T, Yamashita T, Saigusa R, Nakamura K, Noda S, Aozasa N, Toyama T, Takahashi T, Ichimura Y, Sumida H, Tada Y, Sugaya M, Kadono T, Sato S (2015). Increased expression of chemerin in endothelial cells due to Fli1 deficiency may contribute to the development of digital ulcers in systemic sclerosis. Rheumatology (Oxford).

[28] Yamashita T, Asano Y, Taniguchi T, Nakamura K, Saigusa R, Takahashi T, Ichimura Y, Toyama T, Yoshizaki A, Miyagaki T, Sugaya M, Sato S (2016). A potential contribution of altered cathepsin L expression to the development of dermal fibrosis and vasculopathy in systemic sclerosis. Exp Dermatol.

[29] Takahashi T, Asano Y, Nakamura K, Yamashita T, Saigusa R, Ichimura Y, Toyama T, Taniguchi T, Yoshizaki A, Tamaki Z, Tada Y, Sugaya M, Kadono T, Sato S (2016). A potential contribution of antimicrobial peptide LL-37 to tissue fibrosis and vasculopathy in systemic sclerosis. Br J Dermatol.

[30] Taniguchi T, Asano Y, Akamata K, Noda S, Takahashi T, Ichimura Y, Toyama T, Trojanowska M, Sato S (2015). Fibrosis, vascular activation, and immune abnormalities resembling systemic sclerosis in bleomycin-treated fli-1-haploinsufficient mice. Arthritis Rheumatol.

[31] Asano Y, Stawski L, Hant F, Highland K, Silver R, Szalai G, Watson DK, Trojanowska M (2010). Endothelial Fli1 deficiency impairs vascular homeostasis: a role in scleroderma vasculopathy. Am J Pathol.

[32] Saigusa R, Asano Y, Yamashita T, Taniguchi T, Takahashi T, Ichimura Y, Toyama T, Yoshizaki A, Miyagaki T, Sugaya M, Sato S (2016). Fli1 deficiency contributes to the downregulation of endothelial protein C receptor in systemic sclerosis: a possible role in prothrombotic conditions. Br J Dermatol.

[33] Takahashi T, Asano Y, Sugawara K, Yamashita T, Nakamura K, Saigusa R, Ichimura Y, Toyama T, Taniguchi T, Akamata K, Noda S, Yoshizaki A, Tsuruta D, Trojanowska M, Sato S (2017). Epithelial Fli1 deficiency drives systemic autoimmunity and fibrosis: possible roles in scleroderma. J Exp Med.

[34] Sládek NE (2003). Human aldehyde dehydrogenases: potential pathological, pharmacological, and toxicological impact. J Biochem Mol Toxicol.

[35] Wu M, Assassi S (2013). The role of type 1 interferon in systemic sclerosis. Front Immunol.

[36] Christmann RB, Sampaio-Barros P, Stifano G, Borges CL, de Carvalho CR, Kairalla R, Parra ER, Spira A, Simms R, Capellozzi VL, Lafyatis R (2014). Association of Interferon- and transforming growth factor beta-regulated genes and macrophage activation with systemic sclerosis-related progressive lung fibrosis. Arthritis Rheumatol.

[37] Ah Kioon MD, Tripodo C, Fernandez D, Kirou KA, Spiera RF, Crow MK, Gordon JK, Barrat FJ: Plasmacytoid dendritic cells promote systemic sclerosis with a key role for TLR8. Sci Transl Med 2018;10(423):eaam8458. 10.1126/scitranslmed.aam8458. 10.1126/scitranslmed.aam8458 PMC9865429 29321259

[38] Saigusa R, Asano Y, Taniguchi T, Hirabayashi M, Nakamura K, Miura S, Yamashita T, Takahashi T, Ichimura Y, Toyama T, Yoshizaki A, Trojanowska M, Sato S (2018). Fli1-haploinsufficient dermal fibroblasts promote skin-localized transdifferentiation of Th2-like regulatory T cells. Arthritis Res Ther.

[39] Akamata K, Asano Y, Aozasa N, Noda S, Taniguchi T, Takahashi T, Ichimura Y, Toyama T, Sato S (2014). Bosentan reverses the pro-fibrotic phenotype of systemic sclerosis dermal fibroblasts via increasing DNA binding ability of transcription factor Fli1. Arthritis Res Ther.

[40] Akamata K, Asano Y, Yamashita T, Noda S, Taniguchi T, Takahashi T, Ichimura Y, Toyama T, Trojanowska M, Sato S (2015). Endothelin receptor blockade ameliorates vascular fragility in endothelial cell-specific fli-1-knockout mice by increasing fli-1 DNA binding ability. Arthritis Rheumatol.

[41] Matsushita T, Hasegawa M, Hamaguchi Y, Takehara K, Sato S (2006). Longitudinal analysis of serum cytokine concentrations in systemic sclerosis: association of interleukin 12 elevation with spontaneous regression of skin sclerosis. J Rheumatol.

[42] Murata M, Fujimoto M, Matsushita T, Hamaguchi Y, Hasegawa M, Takehara K, Komura K, Sato S (2008). Clinical association of serum interleukin-17 levels in systemic sclerosis: is systemic sclerosis a Th17 disease?. J Dermatol Sci.

[43] Zhou Y, Hou W, Xu K, Han D, Jiang C, Mou K, Li Y, Meng L, Lu S (2015). The elevated expression of Th17-related cytokines and receptors is associated with skin lesion severity in early systemic sclerosis. Hum Immunol.

[44] Yang X, Yang J, Xing X, Wan L, Li M (2014). Increased frequency of Th17 cells in systemic sclerosis is related to disease activity and collagen overproduction. Arthritis Res Ther.

[45] Frantz C, Auffray C, Avouac J, Allanore Y (2018). Regulatory T cells in systemic sclerosis. Front Immunol.

[46] MacDonald KG, Dawson NA, Huang Q, Dunne JV, Levings MK, Broady R (2015). Regulatory T cells produce profibrotic cytokines in the skin of patients with systemic sclerosis. J Allergy Clin Immunol.

[47] Liu ZM, Wang KP, Ma J, Guo Zheng S (2015). The role of all-trans retinoic acid in the biology of Foxp3+ regulatory T cells. Cell Mol Immunol.

[48] Piersma AH, Hessel EV, Staal YC (2017). Retinoic acid in developmental toxicology: teratogen, morphogen and biomarker. Reprod Toxicol.

[49] Toyama T, Asano Y, Miyagawa T, Nakamura K, Hirabayashi M, Yamashita T, Saigusa R, Miura S, Ichimura Y, Takahashi T, Taniguchi T, Yoshizaki A, Sato S (2017). The impact of transcription factor Fli1 deficiency on the regulation of angiogenesis. Exp Dermatol.

[50] Nakahara T, Kido-Nakahara M, Ohno F, Ulzii D, Chiba T, Tsuji G, Furue M (2018). The pruritogenic mediator endothelin-1 shifts the dendritic cell-T-cell response toward Th17/Th1 polarization. Allergy.

[51] Guiducci S, Bellando Randone S, Bruni C, Carnesecchi G, Maresta A, Iannone F, Lapadula G, Matucci Cerinic M (2012). Bosentan fosters microvascular de-remodelling in systemic sclerosis. Clin Rheumatol.

[52] Cestelli V, Manfredi A, Sebastiani M, Praino E, Cannarile F, Giuggioli D, et al. Effect of treatment with iloprost with or without bosentan on nailfold videocapillaroscopic alterations in patients with systemic sclerosis. Mod Rheumatol. 2017; 27(1):110–14. 10.1080/14397595.2016.1192761. 10.1080/14397595.2016.1192761 27310203

[53] Cutolo M, Zampogna G, Vremis L, Smith V, Pizzorni C, Sulli A (2013). Longterm effects of endothelin receptor antagonism on microvascular damage evaluated by nailfold capillaroscopic analysis in systemic sclerosis. J Rheumatol.

[54] Yashiro T, Yamaguchi M, Watanuki Y, Kasakura K, Nishiyama C (2018). The transcription factors PU.1 and IRF4 determine dendritic cell-specific expression of RALDH2. J Immunol.

