# Supplementary figures and images for: Fli1 deficiency suppresses RALDH1 activity of dermal dendritic cells and related induction of regulatory T cells: a possible role in scleroderma
Source: Arthritis Res Ther. 2021 May 8;23:137. doi: 10.1186/s13075-021-02520-z (PMC8106158; doi:10.1186/s13075-021-02520-z)

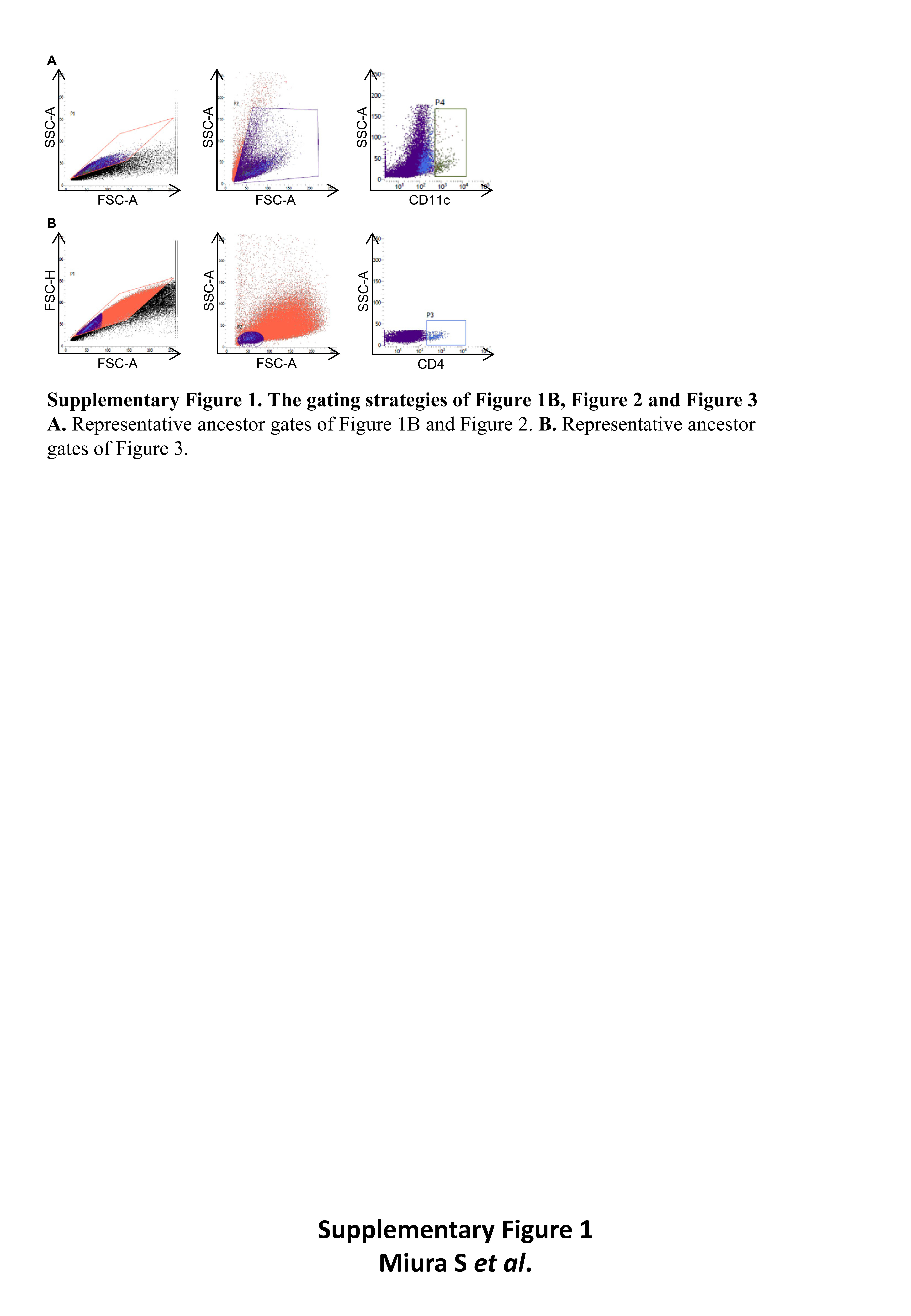

Supplement: Supplementary file 1 — Additional file 1: Supplementary Figure 1. The gating strategies of Figure 1B, Figure 2 and Figure 3. A. Representative ancestor gates of Figure 1B and Figure 2. B. Representative ancestor gates of Figure 3. [file 13075_2021_2520_MOESM1_ESM.jpg]
